# A Common Origin for the Bacterial Toxin-Antitoxin Systems *parD* and *ccd*, Suggested by Analyses of Toxin/Target and Toxin/Antitoxin Interactions

**DOI:** 10.1371/journal.pone.0046499

**Published:** 2012-09-28

**Authors:** Andrew B. Smith, Juan López-Villarejo, Elizabeth Diago-Navarro, Lesley A. Mitchenall, Arjan Barendregt, Albert J. Heck, Marc Lemonnier, Anthony Maxwell, Ramón Díaz-Orejas

**Affiliations:** 1 Departament of Biological Chemistry, John Innes Centre, Norwich Research Park, Norwich, United Kingdom; 2 Department of Molecular Microbiology, Centro de Investigaciones Biológicas-CSIC, Madrid, Spain; 3 Biomolecular Mass Spectrometry and Proteomics Group, Bijvoet Center for Biomolecular Research and Utrecht Institute for Pharmaceutical Sciences, University of Utrecht, Utrecht, The Netherlands; King's College London, United Kingdom

## Abstract

Bacterial toxin-antitoxin (TA) systems encode two proteins, a potent inhibitor of cell proliferation (toxin) and its specific antidote (antitoxin). Structural data has revealed striking similarities between the two model TA toxins CcdB, a DNA gyrase inhibitor encoded by the *ccd* system of plasmid F, and Kid, a site-specific endoribonuclease encoded by the *parD* system of plasmid R1. While a common structural fold seemed at odds with the two clearly different modes of action of these toxins, the possibility of functional crosstalk between the *parD* and *ccd* systems, which would further point to their common evolutionary origin, has not been documented. Here, we show that the cleavage of RNA and the inhibition of protein synthesis by the Kid toxin, two activities that are specifically counteracted by its cognate Kis antitoxin, are altered, but not inhibited, by the CcdA antitoxin. In addition, Kis was able to inhibit the stimulation of DNA gyrase-mediated cleavage of DNA by CcdB, albeit less efficiently than CcdA. We further show that physical interactions between the toxins and antitoxins of the different systems do occur and define the stoichiometry of the complexes formed. We found that CcdB did not degrade RNA nor did Kid have any reproducible effect on the tested DNA gyrase activities, suggesting that these toxins evolved to reach different, rather than common, cellular targets.

## Introduction

The *ccd* and *parD* systems of plasmids F and R1 were the two first proteic bacterial toxin-antitoxin (TA) systems identified [1], [2]. Both TA modules bear antitoxin and toxin genes of small and similar sizes that are organized within an operon. The antitoxin protein of each system interacts with its cognate toxin to neutralise the activity of the toxin, and also leads to the formation of an efficient repressor [3]–[7]. The toxins of the *ccd* and *parD* systems act on different targets: CcdB targets and inhibits DNA gyrase [8], while Kid (identical to PemK) is a specific endoribonuclease that inhibits translation and other RNA-dependent processes [9], [10]. The crystal structures of CcdB [11] and Kid [12] have been solved. In spite of functional differences, comparison of these structures indicated that both toxins share a common structural module [12]. This structural homology prompted an alignment between the CcdB and Kid toxins that was difficult to detect otherwise due to the low similarity in their amino-acid sequences. The antitoxins of the *ccd* and *parD* systems have been reported not to have cross-neutralizing activities on the toxin of the other system [13]. However, alignments between the antitoxins of these systems have been proposed [13], [14] in support of the hypothesis of their common origin. The crystal structure of MazE-MazF (also called ChpAI and ChpAK [15]), the antitoxin and toxin proteins of a system homologous to *parD* found in the chromosome of *Escherichia coli*, has also been solved [14]. MazE and Kis (*parD* antitoxin protein) share a high degree of similarity and the structures of the MazF (in complex [14]) and Kid (antitoxin-free [12]) toxins are very similar. The functional organization of the CcdA and Kis antitoxins is also similar, with an N-terminal region specifically involved in regulation and a C-terminal region more involved in toxin neutralization [7], [13]. These antitoxins share clear homology with the MazE antitoxin that forms a dimer in which the N-terminal region is structured; the C-terminal region of MazE is disorganized in solution and in the dimer make specific contacts with the toxin that lead to its neutralization [5], [14], [16]. This structural information suggests that the toxins and antitoxins of the *parD* and *ccd* systems could interact in a similar way.

In the case of Kid and Kis binding, 4 different interaction sites have been proposed, 3 of them involving the C-terminal region of the antitoxin and the 4^th^ one compromising the N-terminal region of Kis and the toxin [17]. Functional or physical interactions between toxin and antitoxins of homologous TA systems have been previously reported [17], [18]. By native mass spectrometry and NMR spectroscopy, interactions between Kid and MazE antitoxin, that neutralized the activity of the Kid toxin, were analysed. The pattern of interaction and the stoichiometry of the complexes formed (heterotetramers instead of heterohexamers) changed in these interactions. Further structural information on complexes of CcdB and a gyrase fragment [19] and of Kid and its RNA target [20] showed that the RNase and gyrase-binding activities are separated in these toxins which open the possibility of their coexistence in the common ancestor. Taken together, the available information suggests that the *parD* and *ccd* systems could derive from a common ancestor in which the toxins evolved to reach different targets and the antitoxins co-evolved with their toxins to neutralise their activity. Since detailed structural and mechanistic information is now available on these systems the question of whether the common origin of these systems can be traced at the functional level is within experimental reach. Therefore we have analysed the cross activities and interactions between the components of the *ccd* and *parD* systems using purified proteins and *in vitro* assays.

## Results

### RNA cleavage and protein synthesis assays

**Figure 1:**
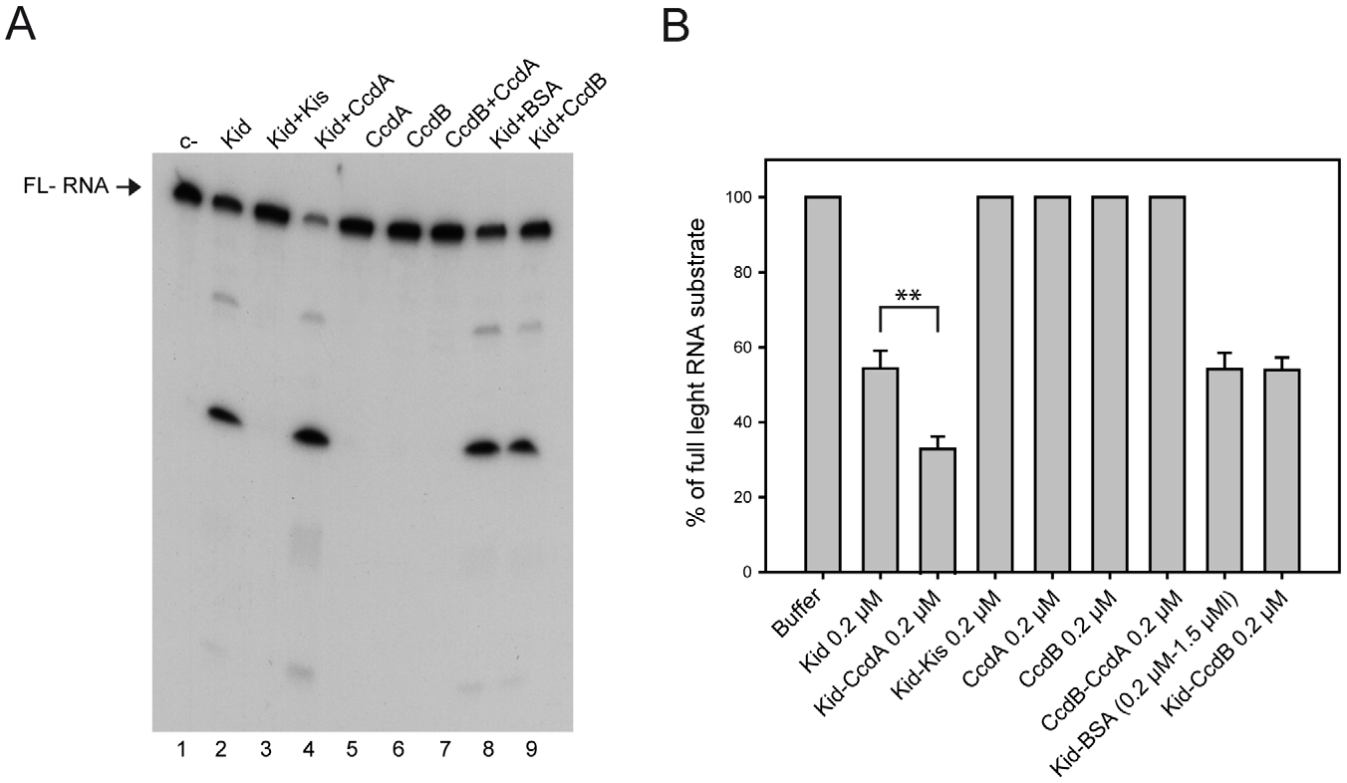
*In vitro* analysis of RNA cleavage by Kid/Kis and CcdB/CcdA. (A) 5′ ^32^P-labelled CopT RNA was incubated for 2 min at 37°C in the presence of Kid or CcdB prior to separation on 8% polyacrylamide gels in the presence of urea. Tracks: 1, C−, untreated full-length (FL) RNA; 2, RNA treated with 0.2 µM Kid; 3, RNA treated with 0.2 µM Kid and 0.2 µM Kis; 4, RNA treated with 0.2 µM Kid and 0.2 µM CcdA; 5, RNA treated with 0.2 µM CcdA; 6, RNA treated with 0.2 µM CcdB; 7, RNA treated with 0.2 µM CcdA and 0.2 µM CcdB; 8, RNA treated with 0.2 µM Kid and 1.5 µM BSA; 9, RNA treated with 0.2 µM Kid and 0.2 µM CcdB. (B) Bar graph representation and quantitative analysis of data in (A): Total RNA (uncleaved and cleaved products) was calculated for each track scanning the different bands using the Quantity One® program (Bio-Rad). RNase activity was calculated as the percentage of full length RNA substrate. The average value for each condition was calculated from three independent experiments. The 100% value indicates absence of RNase activity. The standard deviation is indicated above the different bars. A Student's t-test indicated that the differences between values in lanes 2 and 3, linked with a bracket, were significant (p-value = 0.0067). ** Represents a p-value≤0.01.

The Kid toxin is an endoribonuclease whose action is specifically neutralized by the Kis antitoxin [9], [10]. CopT RNA, the leading region of the messenger of the RepA initiation protein of plasmid R1 that interacts with CopA antisense RNA, is a convenient substrate to assay the RNase activity of Kid. We tested the effects of Kid and CcdB toxins and of Kis and CcdA antitoxins, alone or in combination, on the CopT substrate. Conditions that promote a limiting cleavage of CopT by Kid were used in order to evaluate possible stimulatory effects of CcdA antitoxin that were observed in preliminary experiments. The results (Fig. 1) indicate Kid cleaves CopT giving a major cleavage product and that this activity is prevented by Kis antitoxin. Neither CcdA nor CcdB alone or in combination show any significant RNase activity. Interestingly, it was confirmed that CcdA enhanced significantly the RNase activity of Kid (Fig. 1A and 1B bar 2 and 3, p-value = 0.0067). Compared to the control (Fig. 1B bar 2) neither CcdB nor BSA affected significantly the RNase activity of Kid (p-values = 0.937 and = 0.917 respectively; see two last bars in Fig. 1B). These data suggested that the effect of CcdA could be the consequence of its direct interaction with the Kid toxin rather than due to a protein-dependent stabilization of Kid RNase. In addition we found that CcdA also enhanced the RNase activity of MazF although to a lesser extent than its effect on the RNase activity of Kid (data not shown).

**Figure 2:**
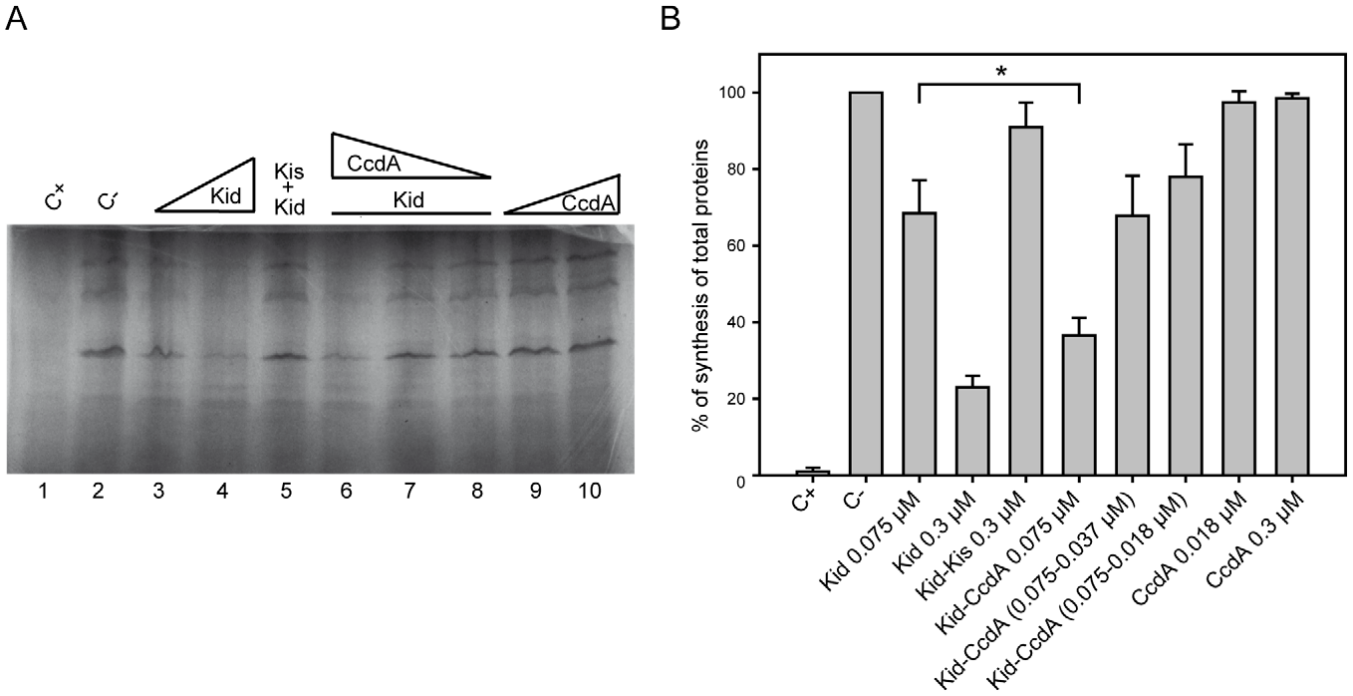
Effects of Kid/Kis and CcdB/A on protein synthesis in *E. coli* cell extracts. (A) Coupled transcription/translation assay with *E. coli* S30 extracts using a luciferase-encoding plasmid as template. ^35^S-labelled products translated in the absence or presence of purified Kid, Kis, CcdB and CcdA proteins were analysed using 15% SDS-PAGE. Tracks: 1, C+ control sample treated with chloramphenicol (30 µg/mL); 2, C−, assay run in the absence of toxin/antitoxin proteins; 3 and 4, effects of adding Kid (0.075, 0.3 µM); 5, neutralisation of Kid by Kis (0.3 µM each); 6, 7, 8, assays run in the presence of Kid (0.075 µM) and CcdA at 0.075, 0.037, 0.018 µM respectively; 9, 10, CcdA alone at 0.018, 0.075 µM. (B) Bar graph representation and quantitative analysis of data in (A). The signals of the labelled proteins were scanned and an integrated value calculated for each condition using a Quantity One informatics program. The percentage of synthesis of total proteins in each track was referred to the value obtained in the untreated sample. The bars above the different values represent the standard deviation calculated from three independent experiments. A Student's t-test indicated that the differences between values in bars linked with a bracket were significant (p-value = 0.0217). * Represents a p-value≤0.05.

Due to its RNase activity, Kid is also a protein synthesis inhibitor and Kis antitoxin specifically abrogates this inhibition. In the next experiment we asked whether the stimulation of the RNase activity of Kid by CcdA could be traced at the level of protein synthesis; for this purpose an assay based on the coupled transcription-translation synthesis of luciferase in cell free extracts of *E. coli* was used (Fig. 2). The effect of CcdA on the activity of Kid was assayed in conditions where Kid gives partial inhibition of protein synthesis (Fig. 2A, Track 3); the partial inhibition mediated by Kid was clear and significantly enhanced by equimolar concentrations of CcdA (Track 6, p-value 0.0217). This enhancement was not observed at lower concentrations of CcdA (Tracks 7 and 8). CcdA at the maximal concentration used, did not show an inhibitory effect (Tracks 10 and 9). CcdB alone or in combination with CcdA did not show an inhibitory effect (data not shown). Chloramphenicol, an inhibitor of protein synthesis, clearly inhibited the assay (compare Tracks 1 and 2). Kid inhibition of protein synthesis, which was partial at 0.075 µM, was practically complete at 0.3 µM (Tracks 3–4) and Kis antitoxin at a Kis∶Kid molar ratio of 1 neutralized this inhibition (Track 5). These results are consistent with the RNase assays and suggested that the enhancement of Kid activities by CcdA is due to a direct interaction of these proteins rather than to an indirect effect (see below). They further show that CcdB does not have either an RNase or a protein synthesis inhibitory potential.

### Stabilisation of the gyrase-DNA cleavage complex

**Figure 3:**
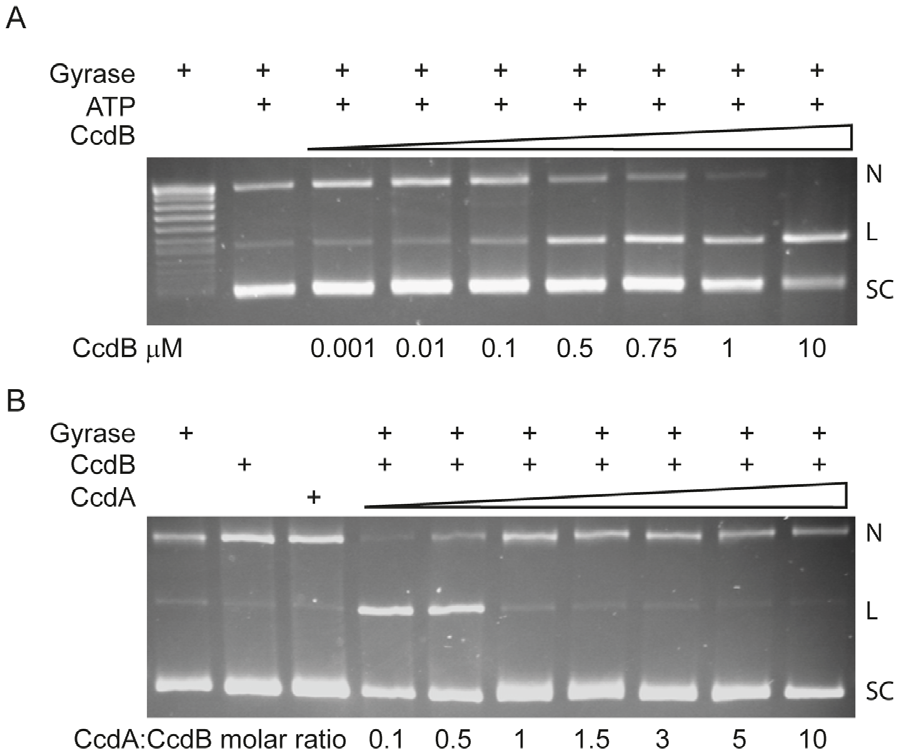
DNA gyrase-mediated cleavage of DNA, stimulated by CcdB. A. Negatively supercoiled pBR322 (3.5 nM) was incubated with 30 nM gyrase, 1.4 mM ATP and various concentrations of CcdB between 0.001–10 µM, as indicated, for 1 h at 25°C. Cleaved DNA was revealed by the addition of SDS and proteinase K. After incubation for 30 mins at 37°C, reactions were stopped with STEB and DNA was subjected to phenol extraction, and samples were analysed on a 1% agarose gel. N, nicked DNA; L, linear DNA; SC, supercoiled DNA. (B) CcdA inhibits the action of CcdB. Negatively supercoiled pBR322 was incubated with gyrase as described in (A) in the presence of 1.4 mM ATP, with 1 µM CcdB and various concentrations of CcdA between 0.1–10 µM as indicated. N, nicked DNA; L, linear DNA; SC, supercoiled DNA.

**Figure 4:**
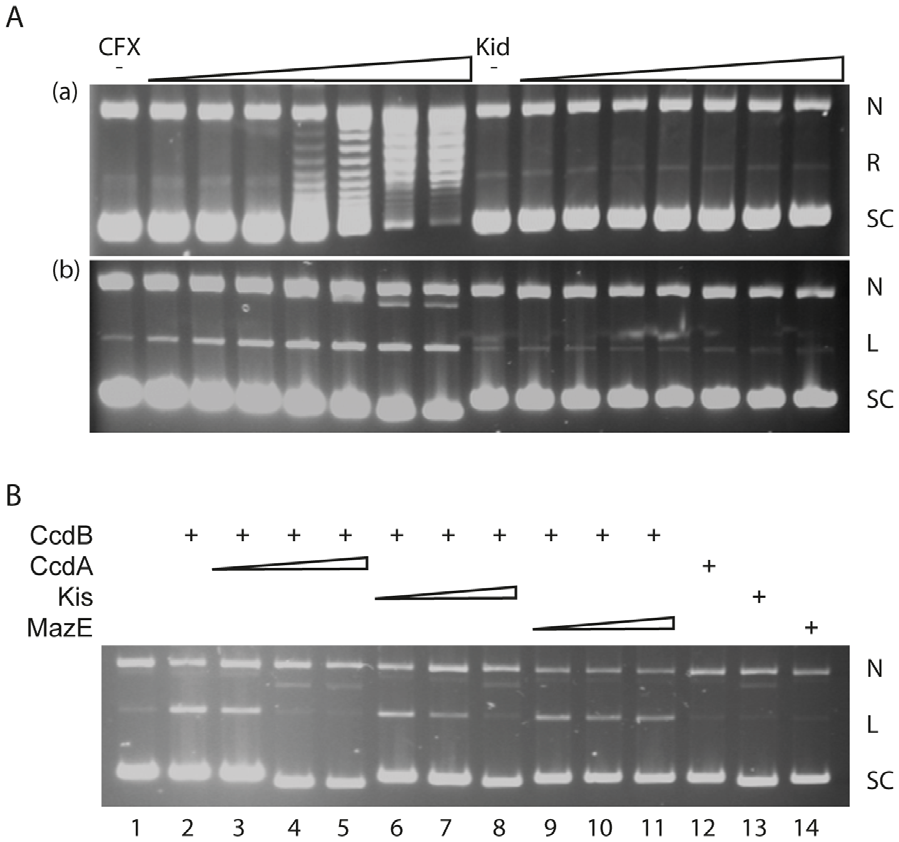
Evaluation of DNA gyrase activity in the presence of different toxins and antitoxins. (A) Kid does not inhibit the action of DNA gyrase. Relaxed pBR322 (21 nM) was incubated with 30 nM gyrase, ATP (1.4 mM) and various concentrations of CFX (ciprofloxacin; 0, 0.1, 0.2, 0.5, 1, 2, 5 and 10 µM) and Kid (0, 0.1, 0.2, 0.5, 1, 2, 5 and 10 µM) as indicated, for 1 h at 37°C. Assays were either (a) stopped or (b) cleaved DNA was revealed by the addition of SDS and proteinase K and incubation at 37°C for 30 mins. Reactions were stopped with 40% sucrose, 100 mM Tris·HCl (pH 7.5), 100 mM EDTA, 0.5 mg/ml bromophenol blue, and samples were subjected to phenol extraction and analysed on a 1% agarose gel (a) run in the absence of ethidium bromide or (b) run in the presence of ethidium bromide (1 µg/mL). N, nicked DNA; L, linear DNA; SC, supercoiled DNA; R, relaxed DNA. (B) Kis, but not MazE, can inhibit the action of CcdB. Relaxed pBR322 (21 nM) was incubated with 30 nM DNA gyrase, ATP (1.4 mM) and various concentrations of CcdB (2 µM), CcdA (1, 2 & 4 µM), Kis (2, 20 & 40 µM) or MazE (2, 20 & 40 µM) as indicated, at 37°C for 1 h. Toxin-antitoxin ratios were 1∶0.5, 1∶1, 1∶2 in Tracks 3–5 and 1∶1, 1∶10, 1∶20 in Tracks 6–8 & 9–11. Antitoxin only controls were at highest concentration used (Tracks 12–14). Cleaved DNA was revealed by the addition of SDS and proteinase K and incubation at 37°C for 30 mins. Reactions were stopped with STEB, and samples were subjected to phenol extraction and analysed on a 1% agarose gel run in the presence of ethidium bromide. N, nicked DNA; L, linear DNA; SC, supercoiled DNA.

The CcdB protein is an inhibitor of *E. coli* DNA gyrase that can stabilise the gyrase-DNA cleavage complex [21]; this inhibition can be prevented by the presence of CcdA (Fig. 3). Fig. 3A shows that, in the presence of ATP, CcdB promotes cleavage of closed-circular DNA to its linear form (up to 50% at 10 µM CcdB). Fig. 3B shows that the addition of CcdA leads to abrogation of cleavage, with the amount of linear DNA returning to background levels (<3%) at molar ratios of CcdA to CcdB of 1 or greater. Other gyrases (e.g. *Mycobacterium tuberculosis* and *M. smegmatis*) are unaffected by CcdB [22], and we have found that neither *E. coli* topo IV nor yeast topo II are affected by CcdB (data not shown). The toxins Kid and its relative MazF were found to have no effect on gyrase supercoiling activity nor were they able to stabilise the cleavage complex under conditions where CcdB and the fluoroquinolone ciprofloxacin could (Fig. 4). Fig. 4A shows that under conditions where ciprofloxacin can inhibit gyrase-catalysed supercoiling (a) and induce DNA cleavage (b), Kid has no effect; the linear band seen in Fig. 4A(b) in the presence of Kid was at background levels (<2%). However, we found that the antitoxin protein Kis could prevent the effect of CcdB in stabilising the gyrase cleavage complex, although MazE was ineffective (Fig. 4B). Specifically we found that, in the presence of CcdA, CcdB-induced DNA cleavage by gyrase was reduced to background levels at a 1∶1 molar ratio of CcdA to CcdB (Fig. 4B, track 4). The presence of a 10∶1 molar excess of Kis to CcdB reduced the level of cleavage to ∼50% of the control (track 7), while a 20∶1 molar excess reduced cleavage to background levels (track 8). The presence of up to a 20∶1 molar excess of MazE over CcdB had no significant effects on the level of cleavage (tracks 9–11).

### Interactions between proteins of the *ccd* and *parD* systems

**Figure 5:**
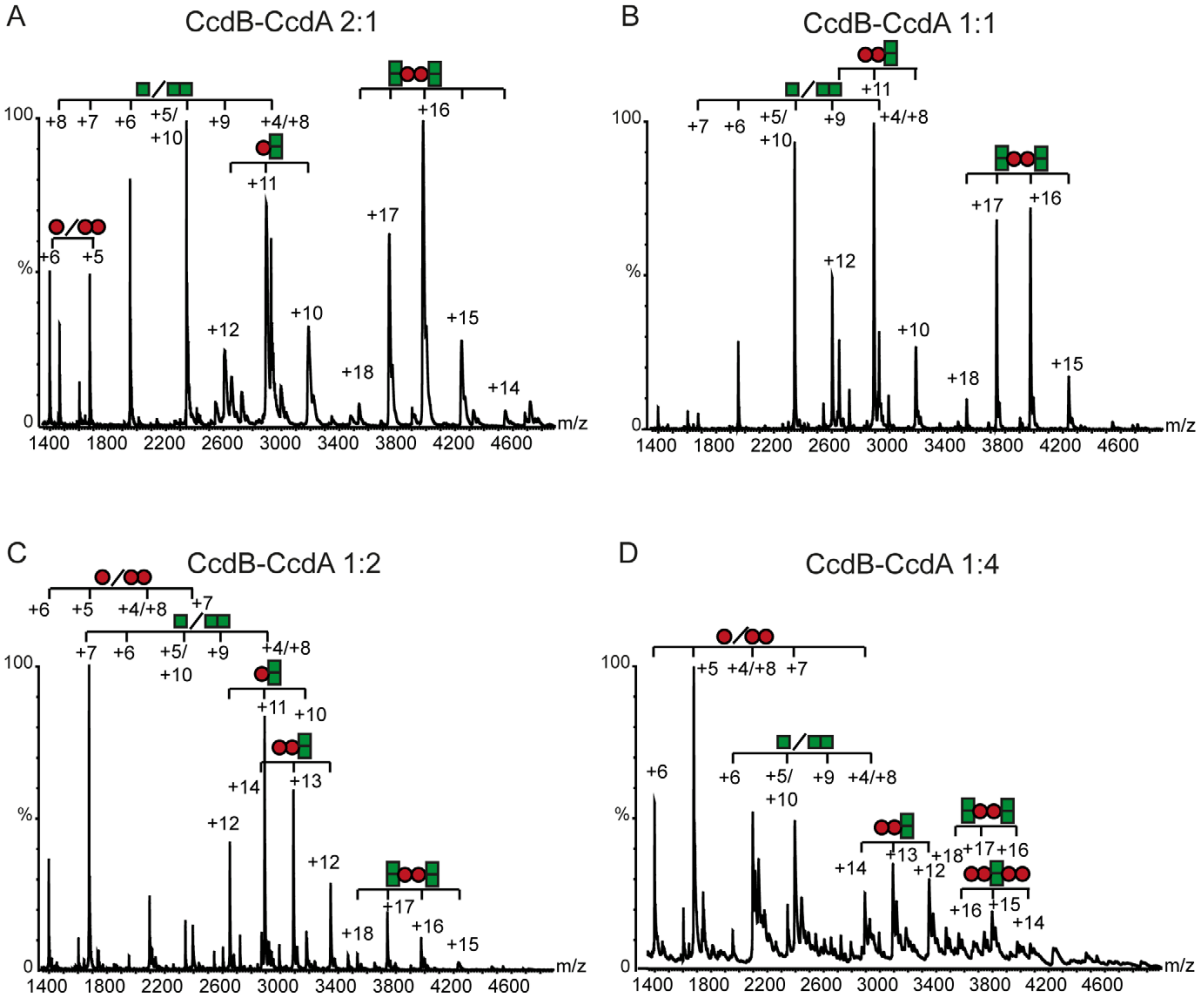
NMS profile of the interactions of CcdB and CcdA proteins at different toxin-antitoxin ratios. (A) Mass spectrum obtained at a CcdB∶CcdA molar ratio of 2∶1. (B) Mass spectrum obtained at a CcdB∶CcdA molar ratio of 1∶1. (C) Mass spectrum obtained at a CcdB∶CcdA molar ratio of 1∶2. (D) Mass spectrum obtained at a CcdB∶CcdA molar ratio of 1∶4. The lowest concentration of protein used was 10 µM. Mixtures of proteins were performed in 100 mM ammonium acetate pH 6.7. CcdB protein is represented with a green square, while CcdA protein is represented with maroon circles. Each complex found is represented with the appropriate combination of squares and circles.

Self and cross-interactions between toxins and antitoxins of the *ccd* and *parD* systems were analyzed by Native Mass Spectrometry (NMS). This is a reliable and sensitive methodology that can be used to detect toxin-antitoxin interaction in solution and to determine the precise stoichiometries of the complexes formed. Kid and Kis interactions complexes formed at different Kis∶Kid ratios have been previously characterised [20], [23]. The most significant species detected were Kis_2_-Kid_2_-Kis_2_-Kid_2_ heterooctamers that are formed at toxin/antitoxin ratios = 1 or in excess of the antitoxin, and Kid_2_-Kis_2_-Kid_2_ heterohexamers that are formed at toxin/antitoxin ratios = 1 or >1. These species are involved respectively in transcriptional regulation of the system and in neutralization of the toxin. The NMS analysis of complexes formed by the CcdA antitoxin and the CcdB toxin at different CcdA/CcdB ratios are shown in Fig. 5. The theoretical and observed molecular masses of the complexes identified are indicated in Table 1. When the CcdB concentration was equal to or in excess of CcdA, soluble trimers, CcdB_2_-CcdA, or hexamers (CcdB_2_-CcdA)_2_ were formed (Fig. 5A–B, Table 1). These complexes have been previously proposed to be involved in the neutralization of the CcdB activity but failed to bind efficiently the promoter-operator region of the system [24]. The analysis indicated that when CcdA was in excess of CcdB, a tetramer, CcdB_2_-CcdA_2_, was found (Fig. 5C–D, Table 1). This complex and multiple forms of it are involved in transcriptional repression of the system [24].

**Table 1:** Theoretical and observed molecular masses of the toxins, antitoxins and their complexes.

His_6_-Kis	Kid	CcdA	CcdB	CcdB_2_-CcdA	CcdA_2_-CcdB_2_	(CcdB_2_-CcdA)_2_	CcdB_2_-HisKis	CcdB_2_-HisKis_2_	Kid_2_-CcdA	Kid_2_-CcdA_2_
E 11220.6	11880.3	8372.3	11706.5	31785.3	40157.6	63570.6	34633.6	45854.2	32132.9	40505.2
O 11225±3	11878±3	8380±6	11710±3	31795±3	40167±6	63582±5	34658±5	45917±7	32162±7	40515±3

Molecular mass is expressed in Daltons.

E: Theoretical molecular mass expected for a protein or complex.

O: Molecular mass observed by Nanoflow Electrospray Ionization Mass Spectrometry. The standard deviation is calculated from at least three different experiments.

**Figure 6:**
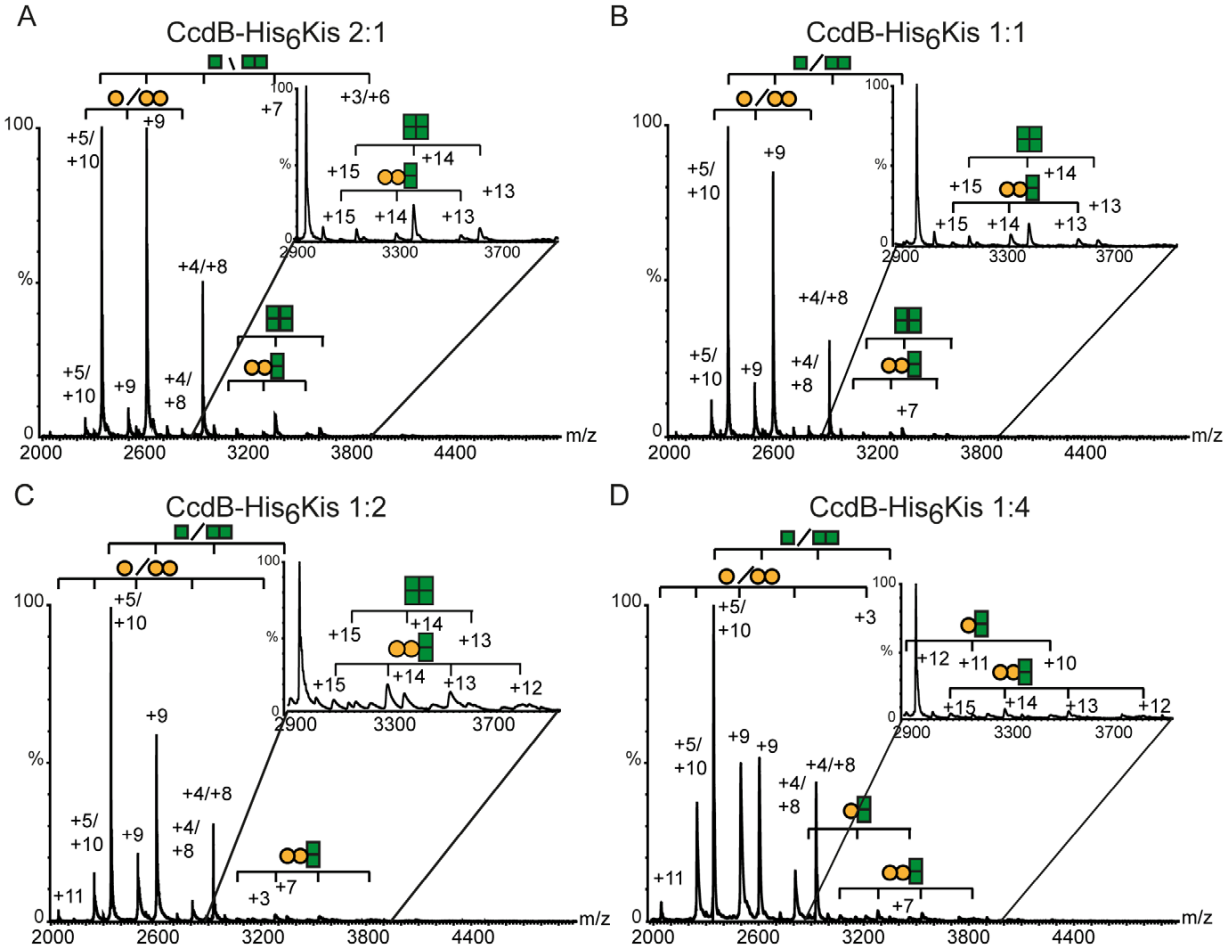
NMS profile of the interactions of CcdB and His_6_Kis proteins at different toxin-antitoxin ratios. (A) Mass spectrum obtained at a CcdB∶His_6_Kis molar ratio of 2∶1. (B) Mass spectrum obtained at a CcdB∶His_6_Kis molar ratio of 1∶1. (C) Mass spectrum otained at a CcdB∶His_6_Kis molar ratio of 1∶2. (D) Mass spectrum obtained at a CcdB∶His_6_Kis molar ratio of 1∶4. All the protein mixtures were performed in 100 mM ammonium acetate pH 6.7. The lowest protein concentration was 10 µM in all cases except the 1∶4 ratio that was 5 µM. CcdB protein is represented with green squares and His_6_Kis with yellow circles. Each complex found is represented with the appropriate combination of squares and circles.

Cross-interactions between toxins and antitoxins of these systems were also analyzed by NMS. The analysis identified complexes formed by CcdB and His_6_Kis at toxin/antitoxins ratios varying from 2∶1 to 1∶4. The charge states corresponding to free His_6_Kis and CcdB proteins were, in all cases, the main species detected. In addition cross-interaction complexes were detected in all cases at the different toxin/antitoxin ratios. When the CcdB toxin was in excess of the His_6_Kis antitoxin, heterotetramers, formed by a dimer of CcdB and a dimer of His_6_Kis (45917±7, Table 1), were detected (Fig. 6A–C, Table 1). When the His_6_Kis concentration was 4 times in excess of CcdB, a trimer formed by a dimer of CcdB and a monomer of His_6_Kis (34654±25, Table 1) was detected (Fig. 6D). The signal corresponding to these cross-complexes was detectable but very much reduced when compared to the one of the CcdA-CcdB complexes (Fig. 5), indicating that cross-interactions were inefficient. This is consistent with the fact that the neutralization of the CcdB anti-topoisomerase activity is far less efficient with the His_6_Kis antitoxin than with CcdA.

**Figure 7:**
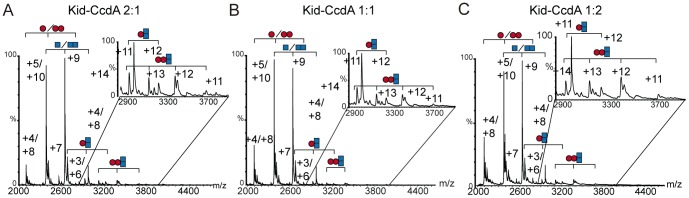
NMS profile of the interactions of Kid with CcdA proteins at different toxin-antitoxin ratios. (A), Mass spectrum obtained at a Kid∶CcdA ratio of 2∶1. (B), Mass spectrum obtained at a Kid∶CcdA ratio of 1∶1.(C) Mass spectrum obtained at a Kid∶CcdA ratio of 1∶2. All the protein mixtures were performed in 100 mM ammonium acetate pH 6.7. The lowest protein concentration was 10 µM in all cases. CcdA protein is represented with maroon circles and Kid protein is represented with blue squares. Each complex found is represented with the appropriate combination of squares and circles.

Similarly cross-interactions between the Kid toxin and the CcdA antitoxin were analyzed at toxin-antitoxin ratios varying from 2∶1 to 1∶2. The charge states corresponding to free Kid and CcdA proteins, were the main species detected but in addition, cross-interaction complexes were detected at the different toxin-antitoxin ratios analysed: trimers Kid_2_-CcdA (32162±7) and tetramers Kid_2_-CcdA_2_ (40557±11) could be detected in all cases (Fig. 7, Table 1). Thus at difference to the complexes formed by His_6_Kis and CcdB, no variations in the stoichiometries of the species formed were found at different toxin-antitoxin ratios, suggesting a distortion in the interaction that could reflect the anomalous stimulatory effect of the CcdA antitoxin on the Kid RNase activity. Again the signal corresponding to the cross-complexes was very much reduced when compared to the one with the CcdA-CcdB complexes.

Consistent with the NMS analysis, His_6_Kis-CcdB interactions could also be detected by Surface Plasmon Resonance (SPR), although with very low signals when compared to the homologous CcdA-CcdB interactions. In the experiment, the CcdB toxin was covalently fixed to the matrix of the chip and His_6_Kis, at different concentrations, was used as the free analyte. The data indicated that this antitoxin interacted with CcdB in a concentration dependent way, with a slow association and dissociation kinetics (Fig. S1). Cross-interactions between Kid and CcdA could not be detected by SPR either with the Kid toxin or the CcdA antitoxin fixed on the chip. All the proteins that were immobilized on the matrix bound efficiently their homologous partners (data not shown). These results and the NMS analysis reported above indicated that the failure to detect CcdA-Kid interactions by SPR reflected that these interactions occurred very inefficiently.

These results showed that physical interactions between the proteins of both systems do exist. It should be stressed that the signals corresponding to the formation of mixed complexes were identified in all the combinations tested but that these were much lower than the ones corresponding to free toxins and antitoxin or to CcdA-CcdB complexes. This strongly suggests that cross-interactions are weaker than self-interactions.

## Discussion

The relationships and differences between *parD*, *chpA*, and *ccd* systems have been reviewed recently [25], [26]. In this work we asked the question of whether a functional crosstalk exists between the protein components of two well-studied toxin-antitoxin systems, *ccd* and *parD*. The toxins of these systems, share a common structural module even if they have different activities (RNase and anti-DNA gyrase respectively). Their structural differences in the common module reflect these different activities. The antitoxins share significant homology but also show clear differences at their N- and C-terminal regions [17], [27].

### Defining toxin-antitoxin interactions

The interactions between toxins and antitoxins have been identified in the *mazEF* and *parD* systems both at the functional and structural levels [14], [17]. Regarding the *ccd* system, CcdB has two sites for the binding of CcdA, which differ in affinity by 6 orders of magnitude [24]. These two sites have implications for the type of CcdA-CcdB complexes formed at different protein ratios. Heterotrimers or heterohexamers are formed in molar excess of the toxin and these play a specific role in neutralization of the toxin; heterotetramers or chains of alternating dimers of CcdA and CcdB are formed in excess of the antitoxin and play a major role in the operon transcriptional regulation [5], [24]. The complexes detected by NMS (Fig. 5) were in agreement with this profile.

In the *parD* system, regulatory complexes are also formed in excess of the antitoxin and a heterooctamer has been identified as the main regulatory species by NMS analysis of repressor-DNA complexes. In excess of the toxin, the main species detected in solution is a heterohexamer. This molecular species binds poorly to the *parD* promoter-operator regions and is more specifically involved in toxin neutralization [23]. The structure of this hexamer has been modelled on the structure of the MazE-MazF heterohexamer previously defined by crystallography [14], [17]. In these heterohexamers the C-terminal region of the antitoxin interact with the terminal helices and the interprotomeric region of the dimeric toxin distorting the action of key residues required for RNA binding and cleavage. An additional interaction region (site 4) that involves contacts between the dimeric toxin and the structured amino-terminal region of the antitoxin could be important to orient the neutralizing interactions of the C-terminal region of the antitoxin with the toxin (see below).

The C-terminal region of CcdA has been shown to be able to neutralize the CcdB toxin [28]. The structural information on this terminal region in complex with CcdB indicates that CcdA contacts the C-terminal helices of the CcdB dimer and invades the inter-protomeric region [5], [24]. As the C-terminal residues of CcdB interact with the major gate of GyrA and the proper positions of the two protomers of CcdB seem to be important/required for additional contacts between CcdB and GyrA [19], this pattern of interaction could lead to inactivation of the CcdB toxin.

### Cross-interactions between toxin and antitoxin of the *parD* and *ccd* families

Differences in the sequence of the homologous toxin and antitoxin could lead to differences in the interaction pattern. Thus, in Kid-MazE complexes assembled in excess of the MazE toxin, two of the four main sites of toxin-antitoxin interactions detected in the Kis-Kid heterohexamer assembled in excess of the Kid toxin, are lost. Furthermore, mass spectrometry analysis indicated that the stoichiometry of the cross-interaction complex is a heterotetramer rather than a heterohexamer [17]. In spite of this, the interaction leads to toxin neutralization probably as the result of the protection of the two symmetrical RNA binding sites in the Kid dimer. This shows that cross-interactions between toxin and antitoxins of homologous systems do not necessarily follow the interaction pattern found between toxin and antitoxins pairs of the same system. In the case of the interaction of CcdA and Kid a change in the interaction pattern results in the enhancement of the Kid activity by the CcdA antitoxin. Functional differences in cross-interactions between a chromosomally-encoded *ccd_O157_* and the *ccd* system of F plasmid *ccd_F_* have also been reported. In this case chromosomally-encoded antitoxin was not able to neutralize the plasmidic toxin while the plasmidic antitoxin neutralized the chromosomal toxin [18]. These cross-interactions remain to be characterized at the structural level.

Kis and CcdA antitoxins show homologies and differences in the N-terminal regions. Kis and CcdA can form dimers but the Kis antitoxin has a LHH fold [17] while CcdA has an RHH fold [27]. The amino acid sequences at the C-terminal regions of both antitoxins differ and in both cases this region is unstructured [17], [27]. These amino acid differences can have important implications in cross antitoxin-toxin interactions. Indeed following hydroxylamine mutagenesis of the *ccdA* gene we could not select a mutant that could neutralized the Kid toxin, which suggest that inhibition of Kid by CcdA, if at all possible, required more than a change [29]. Here we show that unexpectedly, the CcdA protein, can stimulate the RNase activity and protein synthesis inhibitory potential of Kid; As mentioned above, this result could well be the consequence of a distorted CcdA-Kid interaction that makes changes favourable to the residues in Kid important for binding or cleaving the RNA substrate [30]. The fact that the nature of the complexes detected by NMS (mainly CcdA-Kis heterotrimers but also heteroteramers) does not change in response to the relative dosage of the two proteins could well reflect this distortion. In any event further structural information is required to identify the interactions responsible of the enhancement of Kid RNase activity by CcdA.

Functional analysis of Kis-CcdB interactions indicates that Kis can neutralize the anti-topoisomerase activity of the CcdB toxin. This neutralization required a concentration of Kis 20-fold higher than that of CcdA. These results and previous data showing that the basal levels of the antitoxins of the wild-type *ccd* or *parD* systems do not neutralize respectively Kid or CcdB toxins [29], indicate that in practical terms the CcdA and Kis antitoxins are not interchangeable and both systems are functionally independent. A summary of the *ccd* and *parD* toxin-target and toxin-antitoxin interactions is shown in Table 2.

**Table 2:** *ccd* and *parD* toxin-target and toxin-antitoxin interactions.

		CcdB	Kid	MazF
Toxin-target	Gyrase inhibition	√	X	X
	RNA cleavage	X	√	√
Toxin-antitoxin	CcdA	√√	*	*
	Kis	√	√√	√
	MazE	X	√^1^	√√

√ indicates the toxin has the activity or the neutralisation of the toxin by the antitoxin.

X indicates not effect.

* indicates antitoxin stimulates toxin activity.

1 Data from ref [38].

In summary, our data suggest that the toxins of the two systems evolved from a common module to reach different targets and the antitoxins co-evolved to neutralize their activities. Physical and functional interactions of Kis and CcdB and of CcdA and Kid reported could be a weak molecular memory of the common origin of the *ccd* and *parD* systems. The conservation of the structural module common to Kid and CcdB suggests a common origin for these two toxins. However the memory of a common origin was not traced searching cross-activities of the toxins.

## Materials and Methods

### In vitro RNA cleavage assays

The purified 5′-labelled CopT RNA was prepared as described [31]. *In vitro* cleavage reactions were carried out with 1000 cpm of 5′-end-labelled RNAs in 10 mM KCl, 2 mM HEPES (pH 7.8), in the presence of 4 units of RNAsin Ribonuclease Inhibitor (Ambion). The purified Kid and His6-Kis proteins (1 µl) were added and the reactions were incubated for 2 min at 37°C. Reactions were stopped by adding formamide loading buffer and chilling quickly in dry ice. Labelled RNAs were separated on 6% and 8% polyacrylamide gels containing 7 M Urea in TBE (90 mM Tris·Borate, 2 mM EDTA) buffer with different electrophoresis times to maximise the resolution of the products to be visualized.

### Protein synthesis in cell-free extracts

Assays to monitor protein synthesis in *E. coli* cell-free extracts were started by adding 3 µCi of [^35^S]-methionine and 0.4 µl of the test proteins to initial reaction mixtures (10 µl) that contained the following components of the *E. coli* S30 Extract System for Circular DNA (Promega): pBESTluc plasmid DNA (400 ng), 3 µl of S30 extract, 4 µl of S30 premix, and 1 µl of a mixture of amino-acids minus methionine (1 mM) as described [31]. The reactions were incubated for 1 h at 37°C prior to being analyzed using SDS-PAGE (12.5%). Reactions were stopped by adding loading buffer and chilling quickly in dry ice.

### Statistical analysis

The data shown in figures 1 and 2 are means ± standard deviation of each sample analyzed. The differences between the different proteins were analysed with a paired Student's t-test using GraphPad Prism 5 for Mac software. * Represents a p-value≤0.05 and ** a p-value≤0.01.

### Proteins and DNA

Kid and His6-Kis proteins were purified as described [12], and were diluted in 50 mM KCl, 20 mM HEPES (pH 7.8), 100 µg ml^−1^ BSA before use. GyrA and GyrB were purified as previously described [32] and, with supercoiled and relaxed pBR322 DNA, were from Inspiralis (Norwich UK). CcdB was purified from *E. coli* strain B462 (B410 *gyrA462 zei298*::Tn*10*), carrying plasmid pULB2250 [33]. Overnight cultures were diluted 30-fold into fresh TBAmp (100 µg/ml ampicillin) medium [34]. After 4 h growth at 30°C, expression of CcdB was induced by the addition of IPTG to 0.5 mM. Cells were harvested by centrifugation after a further 3 h growth. Cells were resuspended in 50 mM Tris·HCl (pH 7.8), 1 mM EDTA, 1 mM DTT, 150 mM NaCl and 10% glycerol and disrupted in a French pressure cell. Following ultracentrifugation, the supernatant was dialysed against 50 mM Tris·HCl (pH 8.0) and 150 mM NaCl. The solution was then applied to HiPrep Sephacryl S-200 High Resolution gel filtration column (Pharmacia Biotech) and developed with the same buffer. Fractions containing CcdB were pooled and dialysed against 20 mM Tris·HCl (pH 8.0) and applied to a MonoQ HR 5/5 column (Pharmacia Biotech). Proteins were eluted with a 0–0.4 M NaCl gradient. Fractions containing CcdB (eluted at ∼0.15 M NaCl) were pooled and dialysed against 25 mM MOPS (pH 7.0) and applied to a CM-Sepharose column (Pharmacia Biotech). Proteins were eluted with a 0.1–0.3 M NaCl gradient. Fractions containing CcdB (eluted at ∼0.16 M NaCl), were pooled and dialysed against 50 mM Tris·HCl (pH 7.5), 100 mM KCl, 2 mM DTT, 1 mM EDTA and 10% glycerol. CcdB was estimated to be >95% pure by SDS-PAGE and yielded approximately 5 mg of CcdB per litre of cell culture. CcdA was purified from *E. coli* strain SG22623 (MC4100 *cpsB*::*lacZ Δara malP*::*lacI^q^ Δlon-510*) carrying the plasmid pKK223CcdA [3], as described previously [35]. The *E. coli* strains and plasmids were gifts from M. Couturier and L. Van Melderen.

### Gyrase assays

Gyrase-mediated supercoiling and DNA cleavage reactions were carried out as described previously [36]. Gels were photographed using a SynGene Gene Genius Bioimaging System and bands were quantitated using SynGene GeneTools software.

### Native Mass Spectrometry

Native mass spectrometry (NMS) assays were performed as previously described [20]. All mixtures of proteins were carried out in aqueous 100 mM ammonium acetate (pH 6.7). Kid and CcdB proteins were kept in 100 mM ammonium acetate (pH 6.7), whereas Kis and CcdA proteins were at pH 8.0. Samples were introduced in a nanoflow electrospray orthogonal time-of-flight mass spectrometer (Micromass, LCT, Waters, Manchester, UK) previously modified to operate in high mass range and operating in ion positive mode. Nanoflow electrospray voltages were optimized using capillary and cone voltages of 1200–1300 V and 70–80 V, respectively. All spectra were mass calibrated by using an aqueous solution of caesium iodide (25 mg mL^−1^). Data were analysed using MassLynx version 4.0. Different ratios between toxins and antitoxins were tested.

### Surface Plasmon Resonance

The interaction of Kis antitoxin and CcdB toxin was analyzed by Surface Plasmon Resonance (SPR). SPR experiments were performed in HBS-EP (10 mM HEPES, 0.15 M NaCl, 3 mM EDTA, 0.005% Surfactant P20, pH 7.4) in a biosensor Biacore 3000 (Biacore, GE Healthcare). To regenerate the sensor surface, 10 mM Glycine·HCl, pH 2.0 was used. About 3000 RU of CcdB protein were immobilized on a CM5 sensor chip by an amine coupling reaction as recommended by the supplier. Reference surface was prepared in the same manner, except that all carboxyls were blocked in the absence of any ligand. Wild-type His_6_Kis antitoxin was used as soluble analyte in HBS-EP buffer at concentrations ranging from 625 nM to 10 µM. The binding was carried out at 25°C with a flow rate of 15 µl/min. Data were collected for 240 s of association and 120 s of dissociation. Sensograms with different concentrations of analyte were overlaid, aligned and analyzed with BIAevaluation Software 4.1. All data set were processed using a double-referencing method [37].

## Supporting Information

Figure S1 **Dose-dependent interaction of the Kis antitoxin to the CcdB toxin analyzed by SPR.** SPR sensograms corresponding to time course of His_6_Kis-CcdB interactions obtained in a Biacore 3000 flowing different concentrations of the His_6_Kis antitoxin on the toxin immobilized on the chip. Basic operations and analysis were as indicated in Material and Methods.
(PDF)
