# Supplementary figures and images for: A Common Origin for the Bacterial Toxin-Antitoxin Systems parD and ccd, Suggested by Analyses of Toxin/Target and Toxin/Antitoxin Interactions
Source: PLoS One. 2012 Sep 28;7(9):e46499. doi: 10.1371/journal.pone.0046499 (PMC3460896; doi:10.1371/journal.pone.0046499)

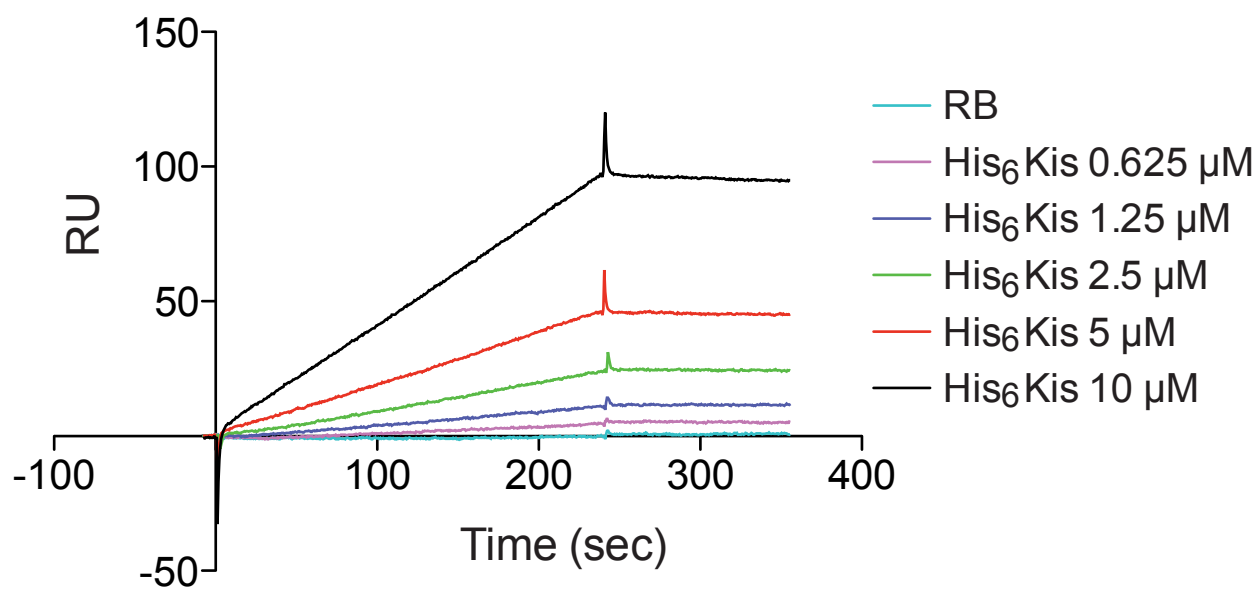

Supplement: Figure S1 — Dose-dependent interaction of the Kis antitoxin to the CcdB toxin analyzed by SPR. SPR sensograms corresponding to time course of His6Kis-CcdB interactions obtained in a Biacore 3000 flowing different concentrations of the His6Kis antitoxin on the toxin immobilized on the chip. Basic operations and analysis were as indicated in Material and Methods. (PDF) [file pone.0046499.s001.pdf]
